# Effects of glucocorticoid receptor activation on gene expression and antiviral responses in Atlantic salmon (*Salmo salar* L.) red blood cells

**DOI:** 10.1186/s13567-025-01605-w

**Published:** 2025-10-07

**Authors:** Thomais Tsoulia, Arvind Y. M. Sundaram, Marit Måsøy Amundsen, Martine Johansen Aardal, Maria Salvador Mira, Frieda Betty Ploss, Randi Faller, Ingvill Jensen, Mona Cecilie Gjessing, Colin Brauner, Maria K. Dahle

**Affiliations:** 1 Departments of Aquatic Animal Health and Analysis and Diagnostics, Norwegian Veterinary Institute, Ås, Norway; 2 Faculty of Biosciences, Fisheries and Economics, Norwegian College of Fishery Science, UiT The Arctic University of Norway, Tromsø, Norway; 3 Department of Medical Genetics, Oslo University Hospital, Oslo, Norway; 4 Department of Biochemistry and Molecular Biology, Institute of Research, Development, and Innovation in Healthcare Biotechnology of Elche, Elche, Spain; 5 Department of Zoology, University of British Columbia, Vancouver, BC Canada

**Keywords:** Atlantic salmon, red blood cells, stress, glucocorticoid receptor, antiviral response

## Abstract

Farmed Atlantic salmon are subjected to different stressors during management routines, leading to prolonged elevated cortisol levels in the blood, a hallmark of chronic stress responses. In this study, we investigate how dexamethasone, an agonist for glucocorticoid receptors (GRs), and cortisol, an endogenous glucocorticoid, regulate gene expression in isolated A. salmon red blood cells (RBCs). Salmonid RBCs express GR genes at high levels. Transcriptional analysis revealed that dexamethasone significantly upregulated 156 genes, and led to > 100-fold induction of the genes *FKBP prolyl isomerase 5* (*fkbp5*), the *Krueppel-like factor 9* (*klf9*), and the *DNA damage-inducible transcript 4* (*ddit4*). The significant upregulation of *fkbp5* persisted for up to two weeks post RBC stimulation, indicating its potential as a biomarker candidate for chronic stress exposure. We compared ex vivo transcriptional responses in RBCs with responses in blood cells from A. salmon injected intraperitoneally with hydrocortisone, and found that *ddit4* may be the most promising biomarker candidate for stress response in vivo. Dexamethasone and cortisol also downregulated antiviral and proteasome genes triggered by double-stranded RNA in RBCs. The results indicate that glucocorticoids have a profound effect on gene expression and putatively alter functions of A. salmon RBCs, and particularly block the antiviral response.

**Supplementary Information:**

The online version contains supplementary material available at 10.1186/s13567-025-01605-w.

## Introduction

Modern salmonid farming is characterized by high-density rearing, short production cycles and intensive handling routines such as mechanical delousing. These practices have been linked to elevated infection pressure and stress responses, posing a significant risk to fish welfare. Farmed Atlantic (A.) salmon (*Salmo salar*) are prone to viral diseases, with stress often identified as the key factor that triggers disease and mortality [1].

Stress initiates the release of catecholamines and glucocorticoids (GCs) into the bloodstream [2, 3]. Unlike in mammals, where the stress response is regulated by the hypothalamic–pituitary–adrenal (HPA) axis, fish regulate the stress response through the hypothalamus-pituitary-interrenal (HPI) axis [2]. Upon stress, corticotropin-releasing factor (CRF) prompts the secretion of adrenocorticotropic hormone (ACTH), which in turn stimulates cortisol production and its release into circulation [4].

Cortisol is the major glucocorticoid in fish and is produced by steroidogenic cells located in the head kidney interrenal tissue [2–4]. The physiological effects of cortisol on target tissues are mediated by its binding to glucocorticoid and/or mineralocorticoid receptors (GR and MR, respectively) [5]. Although cortisol is widely used as an indicator of elevated stress in salmonid farming [2–4], various factors can influence stress intensity and hormonal profile, potentially leading to inaccurate estimation of physiological stress [6].

Chronic stress has been linked to immunosuppression in fish [2, 3, 7]. Experimental studies have shown that clinical disease can be triggered in virus-infected fish by elevated cortisol levels, as shown for the A. salmon viruses infectious pancreatic necrosis virus (IPNV) and salmon gill poxvirus (SGPV) [8, 9]. A third viral disease commonly affecting A. salmon aquaculture is heart and skeletal muscle inflammation (HSMI), caused by Piscine orthoreovirus (PRV-1) [1, 10]. Fish infected with PRV-1 can exhibit increased sensitivity to stress [11, 12], suggesting that HSMI-related mortality may result from a synergistic effect of stress and viral infection [12]. Although increased mortality of virus-infected fish has been associated with stress, the molecular mechanisms underlying this interaction remain relatively uncharacterized.

Fish red blood cells (RBCs) are nucleated, multifunctional cells with diverse physiological and immunological properties [13–16]. Salmonid RBCs have been shown to respond to viruses with robust antiviral responses and play a crucial role in mediating innate immunity [14, 15, 17]. For instance, they have been identified as primary targets of PRV [18].

In this study, we hypothesize that A. salmon RBCs respond to activation of the GR pathway, which can be stimulated by dexamethasone, a synthetic receptor agonist [19], and hydrocortisone. Our aim is to elucidate the effects of stress hormones on RBCs, and to identify novel biomarker candidates indicating the secondary effects of chronic stress. This was obtained through transcriptional analyses of A. salmon RBCs stimulated ex vivo with dexamethasone and hydrocortisone, and blood cells from A. salmon injected with cortisol in vivo.

In addition, we investigated whether dexamethasone and cortisol attenuated the dsRNA-mediated antiviral responses in A. salmon RBCs. To the best of our knowledge, this is the first study on glucocorticoid effects on gene expression and antiviral responses in A. salmon RBCs.

## Materials and methods

### Experimental fish and isolation of red blood cells

The fish (*Salmo salar* L., AquaGen genetic line) used for  RBC isolation were provided by the aquatic research facility at the University of Life Sciences, Ås, Norway, where they were kept in fresh water at 13–15 °C at a density of 6–10 kg/m^3^. The average weight of the sampled A. salmon was approximately 170 g. The fish were anesthetized prior to sampling by bath immersion with Finquel vet (0.5 g/L water) and euthanized using a Finquel vet overdose (1 g/L water) for 2 min. Blood (approx. 1 mL) was collected from the caudal vein of A. salmon in BD vacutainer lithium heparin tubes (VWR International, LLC), and further used for isolation of RBCs.

The heparinized blood was diluted 1:10 in sterile phosphate buffered saline (dPBS). RBCs were isolated by layering on a dPBS-buffered Percoll (GE Healthcare, Uppsala, Sweden) gradient with a bottom layer of 49% Percoll and a top layer of 34%. The diluted blood cells were centrifuged (500 × *g*, 4 °C, 20 min), and the purified RBC pellet was harvested and washed with dPBS, as previously described [18]. Cell quantity and viability (%) were measured using Countess (Invitrogen, Eugene, Oregon, USA). The isolated RBCs were resuspended to a concentration of 2 × 10^7^ cells/mL in Leibovitz’s L15 medium (Life Technologies, Carlsbad, CA, USA) supplemented with fetal calf serum (2%) (Sigma-Aldrich) and gentamicin (50 mg/mL- Lonza Biowhittaker, Walkersville, USA). Culture purity was assessed by examining three areas under a light microscope (approximately 100 cells/area, ≥ 300 cells in total). The culture was considered pure if no more than 2 cells lacked typical RBCs morphology (> 99% culture purity) [14]. The cultures were incubated overnight at 15 °C under constant agitation (225 rpm) prior to experiments.

### Ex vivo stimulation of red blood cells

Dexamethasone and hydrocortisone (both from Sigma-Aldrich Solutions, Darmstadt, Germany) were prepared at stock concentrations of 50 μM and 100 μM, respectively. RBCs of 2 × 10^7^ cells/mL density were treated with either dexamethasone (test concentrations: 1, 10 and 100 μM; *n* = 6 per conc.) or hydrocortisone (test concentrations: 20, 50, 100, 150 μM; *n* = 6 per conc.) and incubated at 15 °C under constant agitation (225 rpm) for 1–14 days before harvest.

Four concentrations of poly (I:C) (Sigma-Aldrich, USA) (25, 50, 100, and 200 μg/mL) and three different harvest points (one, three and seven days) were initially investigated, identifying 50 μg/mL with a three-day stimulation period as optimal for inducing significantly stable dsRNA antiviral responses in A. salmon RBCs.

To investigate the effects of GCs on innate immune responses to poly (I:C), RBCs were pretreated with either dexamethasone or hydrocortisone for 24 h, followed by exposure to 50 μg/mL poly (I:C) and incubated for an additional 72 h prior to harvest (i.e. four days of incubation in total). RBCs treated with either dexamethasone or hydrocortisone for four days, as well as RBCs exposed only to 50 μg/mL poly (I:C) for three days, were also included in the analysis. Untreated RBCs incubated for four days served as controls.

RBCs were harvested by centrifugation (500 × *g*, 14 °C, 10 min). After media removal, the cell pellets were washed with dPBS and lysed in 400 μL MagNA Pure LC Isolation Tissue Kit (Roche Diagnostics, Germany). Lysed cells were stored at −20 °C.

### In vivo experimental trial and blood sampling

Detailed information on the previously published trial can be found in Thoen et al. [8]. In the original trial, the aim was to study the effects of cortisol on fish infected with salmon gill pox virus (SGPV). Briefly, 220 naïve fish with an average body weight of 50 g were divided into four experimental groups. Two groups received intraperitoneal (IP) injection of a depot matrix with hydrocortisone (HC), and two with depot matrix only (Sigma-Aldrich, St. Louis, MO, USA). Two groups were later infected with SGPV, but these groups were not included in the current study.

To reduce effects of handling on endogenous plasma cortisol release prior to blood sampling, fish were first sedated with a low dose of iso-eugenol (2 mg/mL), and finally euthanized with an iso-eugenol overdose (30 mg/mL). At each time point, 5 fish per group were sampled.

Here, blood samples from 5 HC-injected fish and 5 control fish (depot matrix injected only) were studied at two time points: two and four days post-injection (*n* = 20). Blood cell pellets (20 μL) were resuspended in 400 μL lysis buffer from the MagNA Pure Tissue RNA Isolation Kit (Roche) and stored at -20 °C for further analysis.

### RNA isolation

Lysed RBCs (ex vivo experiment) and blood cell pellets (in vivo trial) were homogenized using 5 mm steel beads and a TissueLyser II (Qiagen, Germany) for 3 min at 25 Hz. Total RNA was extracted using MagNA Pure 96 Cellular RNA Large Volume Kit, compatible with the automated MagNA Pure 96 system (Roche Diagnostics, Germany), following the manufacturer’s instructions. RNA was quantified using a Multiskan SkyHigh microplate spectrophotometer (Thermo Fisher Scientific, Norway).

### Illumina RNA sequencing

A total of 40 samples- 20 from the ex vivo RBC stimulation and 20 from the in vivo trial- were sent to the Norwegian Sequencing Centre (NSC, Norway). The RNA quality (RIN > 8) was ensured using Agilent 2100 Bioanalyzer (Agilent, USA) before samples were sent for sequencing. Library preparation was performed using strand-specific TruSeq mRNA-seq Library Prep Kit (Illumina, CA, USA). The experimental groups of the ex vivo experiment included untreated controls (Ctrl, *n* = 5), and RBCs stimulated with poly (I:C) 50 μg/mL (P(I:C), *n* = 5), dexamethasone 100 μM (Dex, *n* = 5), or a combination of dexamethasone 100 μM and poly (I:C) 50 μg/mL (DexP, *n* = 5). For the in vivo trial, the experimental groups included mock controls (*n* = 5 at day two; *n* = 5 at day four) and HC-injected fish (*n* = 5 at day two; *n* = 5 at day four). The libraries from ex vivo and in vivo experiments were pooled separately, and sequenced on one lane of Illumina NovaSeq S4 flow cell to obtain 150 bp paired-end reads. The raw sequencing data are available in BioProjects PRJNA1042786 and PRJNA1042788, respectively.

### Bioinformatic processing and statistical analysis

Raw sequence data (Fastq files) were processed to trim/remove adapter and low-quality sequences using BBDuk tool in BBMap v.38.18 suite (parameters: ktrim = r, k = 23, mink = 11, hdist = 1, tbo, tpe, qtrim = r, trimq = 15, maq = 15, minlen = 36, forcetrimright = 149) [20]. The mapping of cleaned reads to the *Salmo salar* genome (NCBI GCF Ssal_v3.1) was performed using the HISAT2 v.2.2.1 (parameters: –rna-strandness RF) [21]. To estimate the number of reads and aligning against the reference genes in NCBI GCF_905237065.1 annotation, FeatureCounts v.1.4.6-p1 (parameters: -p -s 2) was used [22]. Initial raw data analysis was performed using SARTools v.1.7.4 and R v.4.1.1 [23, 24]. Normalization and differential expression between groups and against the control were performed using DESeq2 v.1.34.0, separately for the ex vivo and in vivo experiments [25]. The annotation tables were cleaned using normalized count reads > 10 as a cutoff, to omit genes with zero or low counts. Adjusted *p*-value (padj) was calculated using Benjamin-Hochberg (BH) correction and genes with padj below 0.05 were considered as differentially expressed genes (DEGs). To assess the variability of the ex vivo and in vivo samples within each experimental condition, we performed principal component analysis (PCA) (Additional files 1 and 2 correspond to the ex vivo samples, and Additional file 3 corresponds to the in vivo samples). One of the control samples from the ex vivo stimulations exhibited strong deviation along the first and second principal components, and the entire experiment was omitted from further RNA-seq analysis (final *n* = 4). RBCs (*n* = 4) showed wide dispersion along the first principal component, but they formed two major clusters, with RBC replicates from the same condition pairing together.

For gene regulation, upregulated features with less than twofold change and downregulated features with higher than 0.5-fold change in expression (0.5 < fold change < 2) were filtered out. STRING Database v.12.0 was used for gene ontology (GO) and Kyoto Encyclopedia of Genes and Genomes (KEGG) enrichment analysis with 0.05 as *p*-value cutoff, BH adjusted [26]. To compare the gene expression profile of RBCs associated with different functional groups across treatments, heatmaps were generated using RStudio v4.3.0.

### qPCR analysis and statistics

Reverse transcription was performed on six RBC experiments (six fish; *n* = 6) from the ex vivo stimulations, using 200 ng total RNA per sample. The RNA was incubated with gDNA Wipeout Buffer (Qiagen) at 42 °C for 2 min to remove genomic DNA, followed by cDNA synthesis using the QuantiTect Reverse Transcriptase Kit (Qiagen). Quantitative PCR (qPCR) was conducted in duplicate on cDNA corresponding to 5 ng RNA input. Gene expression analysis was performed using SsoAdvanced Universal SYBR Green Supermix (BIO-RAD Laboratories, USA) and 400 nM specific A. salmon primers (listed in Table 1). Newly designed primer efficiency, including *FKBP prolyl isomerase 5* (*fkbp5*), was tested using a dose–response standard curve. The amplification ran for 40 cycles of 95 °C/15 s, 60 °C/30 s in a CFX384 Touch Real-Time PCR Detection System (BIO-RAD Laboratories, USA). The melting curves were analyzed to confirm the presence of a single amplicon and absence of primer-dimer formation. The gene expression relative to untreated controls was determined by the 2^−ΔΔCt^ normalization method [27]. The analysis of RT-qPCR data was performed in GraphPad Prism 9, and significant differences were estimated using one-way ANOVA and Dunnett’s test when comparing treated groups with untreated controls, or two-way ANOVA and Tukey’s test when comparisons involved both controls and poly(I:C)-treated RBCs as a reference.

**Table 1:** **Primers used for RT- qPCR analysis**.

Primer	Primer sequence (5’ → 3’)	Accession #
*ef1ab*	Fwd- TGCCCCTCCAGGATGTCTAC	Q9DDK2
	Rev- CACGGCCCACAGGTACTG	
*mx1*	Fwd- GATGCTGCACCTCAAGTCCTATTA	BT043721.1
	Rev- CACCAGGTAGCGGATCACCAT	
*isg15*	Fwd- ATATCTACTGAACATATATCTATCATGGAAACTC	BT048733
	Rev- CCTCTGCTTTGTTGTGGCCACTT	
*fkbp5*	Fwd- TGCTGAGCTTCAAAGGGGAG	BT048177.1
	Rev- AGAGAAGGTAGGTCTGCCTCA	

## Results

### Effects of glucocorticoids on the transcriptional profile of A. salmon red blood cells ex vivo and blood cells in vivo

#### *Effects of dexamethasone on the transcriptional profile of A. salmon red blood cells compared to unstimulated controls *ex vivo

Transcriptional analysis of purified A. salmon RBCs in their resting state revealed the expression of genes encoding proteins involved in stress hormone responses in mammals. These include transmembrane GRs and accessory molecules which are implicated in the regulation of glucocorticoid-induced signaling pathways (Table 2). The normalized transcript reads (ntr) of two commonly used housekeeping genes, *elongation factor 1a* and *1b* (*ef1a* and *ef1b*), are also included in Table 2 for comparison. Four GR genes were found to be expressed.

**Table 2:** **Normalized transcript reads (ntr) of genes encoding glucocorticoid receptors (GR) and accessory proteins implicated in regulation of glucocorticoid-induced signaling pathways in A. salmon RBCs in resting state (*n* = 4).**

Gene reference	Gene ID	Gene name	Control (ntr)
LOC106604224	*gr (ssa05)*	*Glucocorticoid receptor (ssa05)*	1511
LOC106567492	*gr (ssa13)*	*Glucocorticoid receptor (ssa13)*	1082
LOC100380779	*gr (ssa04)*	*Glucocorticoid receptor (ssa04)*	1556
LOC106612223	*gr (ssa09)*	*Glucocorticoid receptor (ssa09)*	1612
LOC106584263	*gmeb1*	*Glucocorticoid modulatory element-binding protein 1*	3260
LOC106572177	*gmeb2*	*Glucocorticoid modulatory element-binding protein 2*	1138
LOC106590407	*sgk3*	*Serum/glucocorticoid regulated kinase family member 3*	1236
LOC100306837	*glci1*	*Glucocorticoid-induced transcript 1 protein 1*	1520
Housekeeping genes			
LOC100136525	*ef1a*	*Elongation factor 1 alpha*	215
LOC100195925	*ef1b*	*Elongation factor 1 beta*	625

For comparison, transcript levels of the* elongation factor 1 alpha* and* beta* (*ef1a* and *ef1b*) housekeeping genes are also given.

^*^GR variants identified in chromosomes 4 (ssa04), 5 (ssa05), 9 (ssa09) and 13 (ssa13) of A. salmon genome.

Differential gene expression analysis was performed to examine the effects of dexamethasone on the transcriptional profile of RBCs, compared to the unstimulated controls. The GR agonist dexamethasone significantly induced 156 genes and reduced transcript levels of 6 genes 24 h after addition to RBCs ex vivo. Among the upregulated DEGs, 12 genes exhibited a 40–600-fold increase, with *Krueppel-like factor 9* (*klf9*) showing the most pronounced upregulation (600-fold change) (Table 3). In addition to *klf9*, the *DNA damage-inducible transcript 4 protein* (*ddit4*) and *fkbp5* genes also demonstrated substantial upregulation, with over 200-fold increases. The *elongation of very long chain fatty acids protein 4* (*elovl4*) gene, which is involved in regulation of polyunsaturated fatty acid (PUFA) biosynthesis in fish [28], was also significantly induced, with an approximate 90-fold increase in response to dexamethasone. Among the downregulated genes, *phosphatidylinositol 4-phosphate 5-kinase-like protein 1* (*pip5kl1*) and *sorting nexin 7* (*snx7*), which exhibited 0.4-fold and 0.5-fold decrease, respectively, have been involved in intracellular protein trafficking via phosphatidylinositol signaling system in mammals [29, 30], however, their function in teleost remains poorly explored. The lists of all upregulated and downregulated DEGs in response to dexamethasone are provided in Additional files 4 and 5, respectively.

**Table 3:** **Normalized transcript reads (ntr) of the twelve most highly induced genes in response to dexamethasone (100 μM) in A. salmon RBCs**.

Gene reference	Gene name	Control		Dexamethasone 100 μM		Fold-change (ctrl vs dex.)
		Mean (ntr)	SD	Mean (ntr)	SD	
LOC106585360	*klf9*	2	1.29	1157	263.59	600.62
LOC106592479	*ddit4*	39	9	10,961	2281.20	268.21
LOC100196052	*fkbp5*	32	14.08	7557	3121.97	218.95
LOC106563189	*inpp5k*	1	0.96	154	101.48	138.5
LOC106564638	*elovl4*	86	45.04	10,370	5658.98	92.46
LOC106563188	*smtna*	3	2.75	258	217.35	74.12
LOC106577395	*ddit4-like*	51	23.62	3499	968.37	66.62
LOC106590608	*mtrr*	455	108.44	28,134	2983.65	59.14
LOC123732461	*prelid3a*	1	1.26	83	47.52	46.25
LOC106569334	*higd1a*	92	9.67	3945	317.14	41.90
LOC106587747	*map1b-like*	5	2.83	238	142.61	41.45
LOC106575072	*cdn1b*	2	2.38	138	66.09	41.45

Data represent the mean normalized values (Mean) and standard deviation (SD) of four biological replicates (n = 4) per group. Fold-change values were calculated relative to untreated controls (ctrl vs dex.).

### *Transcriptional responses of fkbp5 gene in A. salmon red blood cells exposed to dexamethasone and hydrocortisone ex vivo*

**Figure 1:**
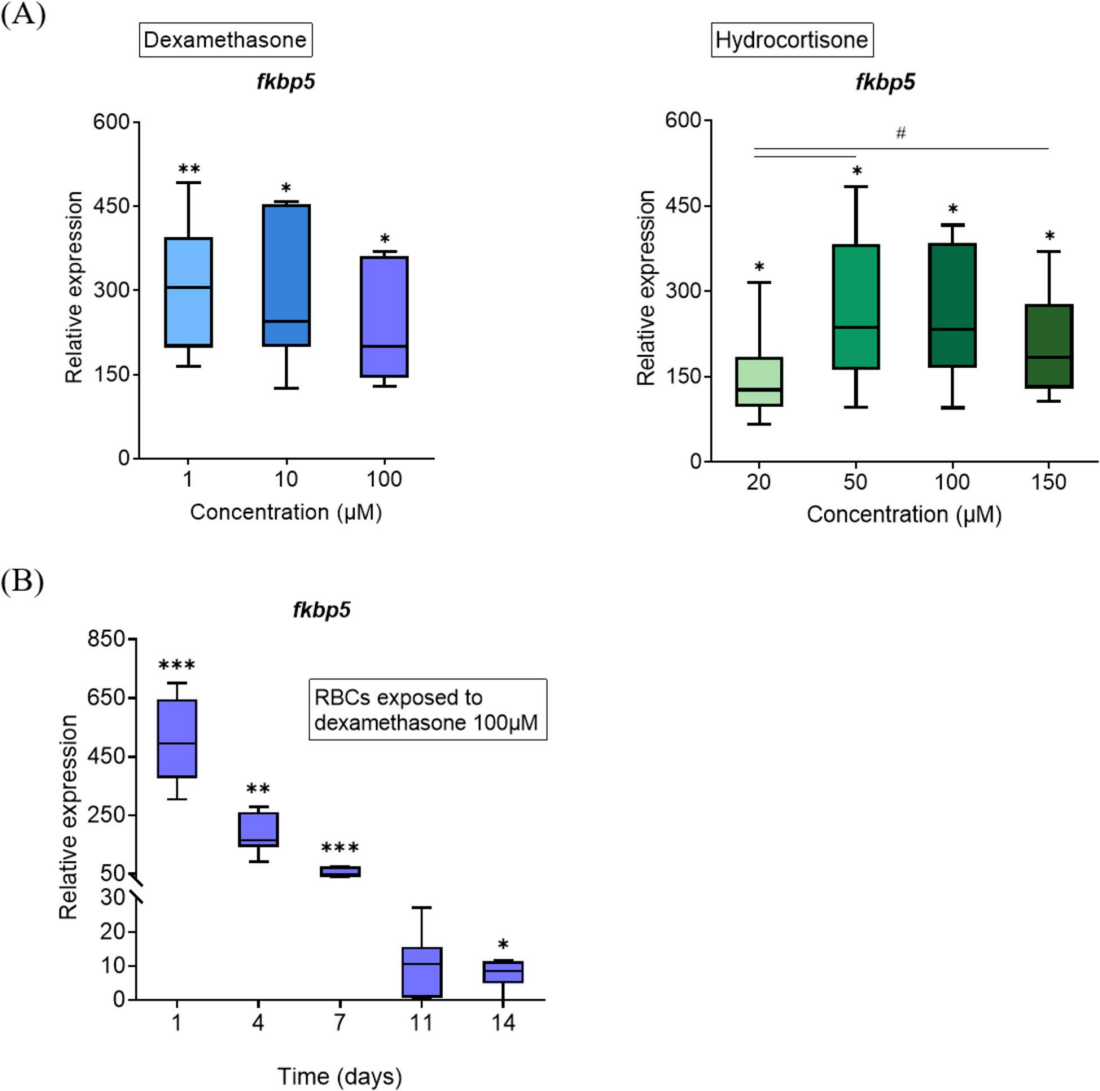
**Effects of dexamethasone and cortisol on the transcriptional profile of *FKBP prolyl isomerase 5*** (*fkbp5*) in A. salmon red blood cells, based on RT qPCR (*n* = 6). A Box plot showing relative expression of *fkbp5* in A. salmon RBCs four days post-stimulation with dexamethasone and hydrocortisone. Error bars represent standard deviation in each plot. *: *p* < 0.05 relative to the control; **: *p* < 0.01 relative to the control; #: *p* < 0.05 between the experimental conditions. B **Box plot showing relative expression of** ***fkbp5*** in A. salmon RBCs stimulated with 100 μM dexamethasone over 14 days. Data were analyzed using paired t-test for the treated RBCs of each day, compared to their respective untreated controls (*n* = 6). Error bars represent standard deviation in each plot. *: *p* < 0.05 relative to the control; **: *p* < 0.01 relative to the control; ***: *p* **< 0.0005.**

The *fkbp5* gene was further investigated as a potential biomarker for chronic stress in RBCs. The relative expression of *fkbp5* in RBCs after a four-day exposure to different concentrations of dexamethasone (1–100 μM) and hydrocortisone (20–150 μM) was assessed using quantitative PCR (Figure 1A). The *fkbp5* was significantly upregulated across all tested concentrations of both dexamethasone and hydrocortisone, showing a > 100-fold increase relative to the controls. Dexamethasone induced high *fkbp5* expression, with approximately 200–300-fold increase (Figure 1A, left panel). In contrast, the lowest concentration of hydrocortisone tested (20 μM) was associated with the lowest *fkbp5* expression. Hydrocortisone at concentrations of 50 and 100 μM, levels often indicative of stressed fish in vivo [31], resulted in an approximate 250-fold increase in *fkbp5* expression, a similar effect observed with 10 μM dexamethasone (Figure 1A, right panel). The highest concentrations of dexamethasone (100 μM) induced a 200-fold upregulation of *fkbp5*, consistent with our transcriptomic findings (Table 3). A similar 200-fold increase was also observed for the highest concentration of hydrocortisone (150 μM) (Figure 1A).

The relative expression of *fkbp5* was monitored over 14 days after the activation of the glucocorticoid receptor pathway by 100 μM dexamethasone in RBCs. The *fkbp5* showed a > 500-fold upregulation just after 24 h of exposure (Figure 1B). Although gene expression gradually decreased over time, a significant 50-fold upregulation was still observed after seven days of exposure. By days eleven and fourteen, *fkbp5* exhibited approximately a tenfold upregulation in dexamethasone-stimulated RBCs.

#### Differential expression analysis of whole blood of Atlantic salmon injected with hydrocortisone compared to sham controls

Transcriptional analysis of A. salmon blood cells was performed at two and four days post IP injection of hydrocortisone, compared to sham controls in vivo. These results were used to determine whether elevated cortisol levels induce transcriptional changes in vivo similar to those observed in RBCs stimulated with dexamethasone ex vivo.

**Figure 2:**
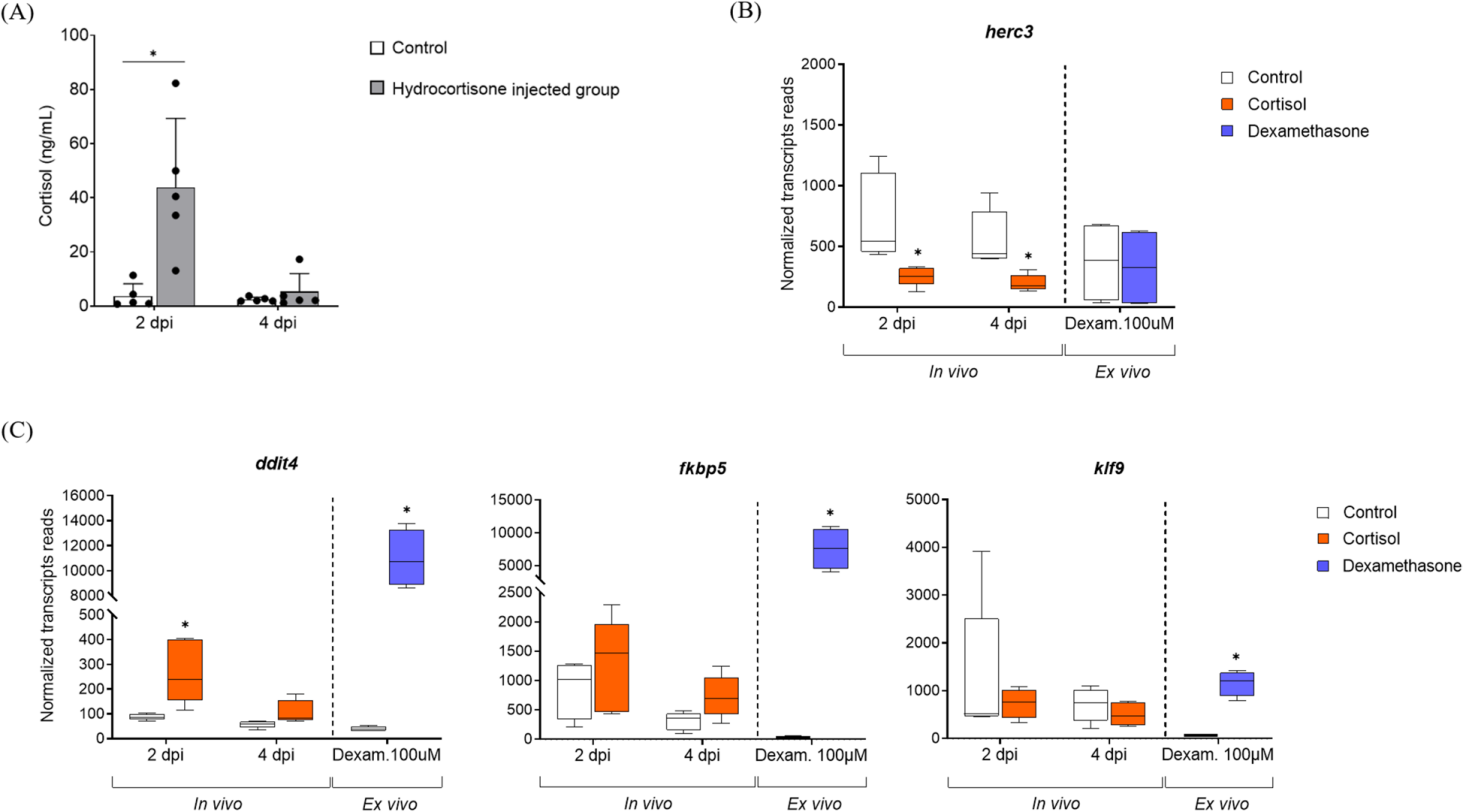
**Transcriptional responses in whole blood of A. salmon two and four days post-injection (dpi) of hydrocortisone, based on RNA-seq data.** **A** **Bar graph showing cortisol levels in blood plasma of A. salmon detected two and four days post-injection, as previously shown by Thoen et al. [**8**]. B Box plot showing transcriptional profile of the** ***E3 ubiquitin-protein ligase HERC3*** **(*herc3*) gene and C the *DNA damage-inducible transcript 4 protein*** **(*ddit4*), *FKBP prolyl isomerase 5*** **(*fkbp5*)** **and *Krueppel-like factor 9*** **(*klf9*)** **genes in blood cells of A. salmon two and four days post-hydrocortisone injection in vivo and in RBCs four-days post-stimulation with dexamethasone ex vivo. RBCs ex vivo: *n* = 4. Whole blood in vivo: *n*** = 5. Error bars represent standard deviation in each plot. *: *p* < 0.05 compared to the controls.

Differential gene expression analysis was performed using a cutoff of > twofold change for upregulated genes and < 0.5 for downregulated genes. In response to elevated cortisol levels two days post-injection, 3 genes were significantly upregulated, while 11 were significantly downregulated in A. salmon blood cells (Additional files 6 and 7, respectively). By day four, a new set of 5 genes were upregulated and 11 genes were downregulated (Additional files 8 and 9, respectively). Only the *E3 ubiquitin-protein ligase HERC3* (*herc3*) gene was significantly suppressed on both day two and four post-injection. The expression level of the *herc3* gene was not affected in the ex vivo stimulation of RBCs with dexamethasone (Figure 2B).

The *ddit4* gene was significantly induced both in RBCs after ex vivo stimulation with dexamethasone, and in blood cells two days post-hydrocortisone injection, when the cortisol levels peaked (Figure 2C). In contrast, the *klf9* and *fkbp5* genes, which exhibited the greatest transcriptional differences in stimulated RBCs relative to the controls, were not significantly induced in blood cells in vivo (Figure 2C). Most of the genes that were significantly upregulated by hydrocortisone in whole blood of A. salmon were not found to be expressed in cultured RBCs (zero normalized transcript count reads), suggesting that their activation may occur in other blood cell types, such as leukocytes. In addition, there was no overlap between the downregulated DEGs observed in blood cells and those in dexamethasone-stimulated RBCs.

### Transcriptional analysis of RBCs stimulated with either dexamethasone, poly (I:C) or dexamethasone and poly (I:C) compared to unstimulated controls ex vivo

**Figure 3:**
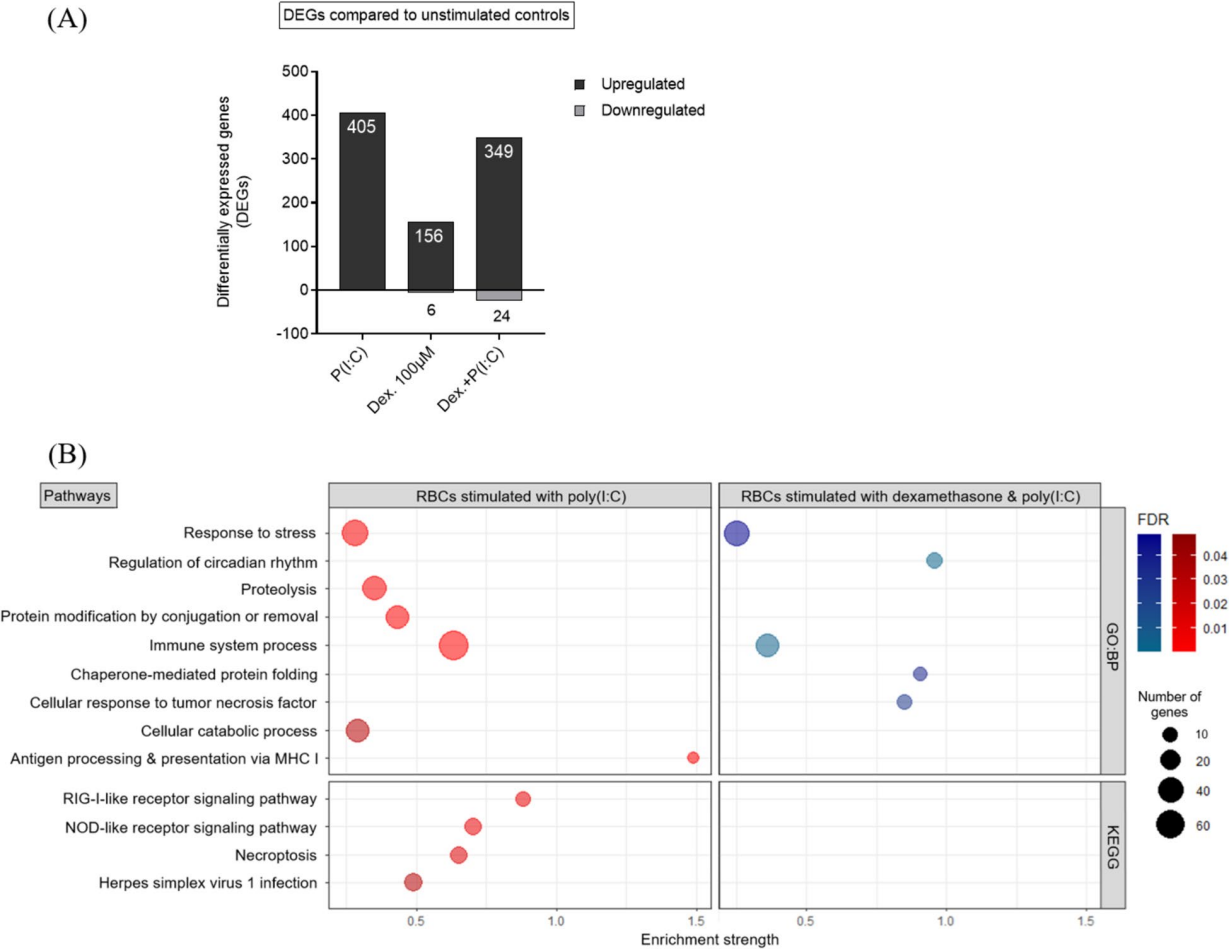
**Transcriptional analysis of A. salmon RBCs stimulated either with 100 μM dexamethasone, 50 μg/mL poly (I:C) or 100 μM dexamethasone and 50 μg/mL poly (I:C), compared to unstimulated controls ex vivo, based on RNA-seq. A Differential gene expression analysis of A. salmon RBC exposed to dexamethasone, poly (I:C), and their combination, compared to their unexposed controls. B Enriched Gene Ontology (GO) terms within the “Biological Process” (GO: BP) and Kyoto Encyclopedia of Genes and Genomes (KEGG) pathway datasets. Only functional groups and pathways with FDR (adjusted *p* value) ≤ 0.05 were considered significant. GO: BP (top) and KEGG pathways (bottom) enriched in RBCs exposed to poly (I:C) (red), and dexamethasone and poly (I:C) (blue).**

RBCs from four A. salmon stimulated with either dexamethasone, poly (I:C) or dexamethasone and poly (I:C), along with unstimulated controls, were analyzed by RNA-seq. Differential gene expression analysis was performed to examine the effects of dexamethasone, poly (I:C) or dexamethasone and poly (I:C) on the transcriptional profile of RBCs, compared to the unstimulated controls (Figure 3A). In addition, to determine the biological processes and signaling pathways to which DEGs of stimulated RBCs belonged, gene ontology (GO) for Biological Process (GO:BP) and Kyoto Encyclopedia of Genes and Genomes (KEGG) pathway enrichment analyses were performed (Figure 3B). Given the limited number of downregulated genes, only the upregulated DEGs of RBCs stimulated with poly (I:C) alone or dexamethasone and poly (I:C) were categorized into functional groups (Figure 3B).

### Effects of dexamethasone and hydrocortisone on poly (I:C)-induced antiviral responses in A. salmon red blood cells ex vivo analyzed by RT- qPCR

The mRNA levels of *myxovirus resistance 1* (*mx1*) and *ISG15 ubiquitin-like modifier* (*isg15*) genes were analyzed in RBCs stimulated with poly (I:C) at concentrations of 25, 50, 100 and 200 μg/mL, following incubation for one, three and seven days. No significant gene induction was observed on day one at any concentration. The *mx1* gene was significantly upregulated by day three post-stimulation, exhibiting a similar response pattern at 50, 100 and 200 μg/mL poly (I:C), with an approximate 20-fold increase relative to the controls. Peak expression occurred on day seven, particularly at 50 μg/mL poly (I:C), where changes in *mx1* gene expression showed a significant 50-fold upregulation, relative to the controls (Additional file 10, Figure 1A). The expression of *isg15* was generally higher than *mx1*, with approximately 70-fold and 200-fold increases in response to 50 and 200 μg/mL poly (I:C) on days three and seven, respectively (Additional file 10, Figure 1B). Based on these observations, along with the substantial variation in responses among replicates on day seven, a three-day exposure of RBCs to 50 μg/mL poly (I:C) was selected as the optimal ex vivo stimulation for eliciting significant antiviral responses.

**Figure 4:**
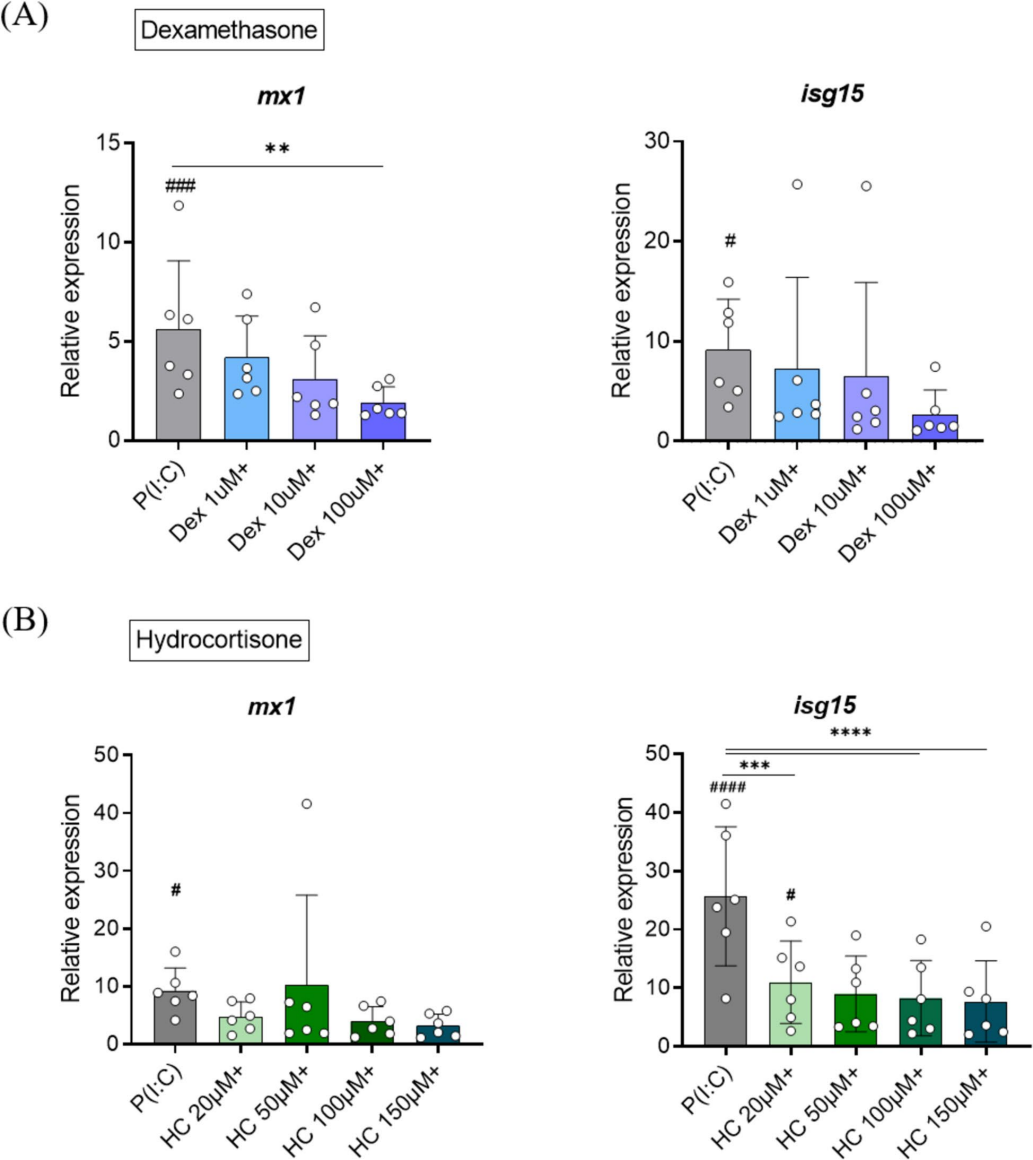
**Effects of dexamethasone or hydrocortisone on antiviral responses of A. salmon RBCs to poly (I:C), based on RT qPCR (*n* = 6). A Scatter plot with bars showing the expression levels of** ***myxovirus resistance 1*** **(*mx1*) and *ISG15 ubiquitin like modifier*** (*isg15*) genes in response to dexamethasone (blue), and B hydrocortisone (green) in A. salmon RBCs ex vivo. Co-stimulation with poly (I:C) is shown with a “ + ”. Error bars represent standard deviation in each plot. #: *p* < 0.05; ####: *p* < 0.0001 compared to the unstimulated controls. *: *p* < 0.05; **: *p* < 0.01; ***: *p* < 0.0005; ****: *p* **< 0.0001 compared to poly (I:C) alone.**

The expression levels of *mx1* and *isg15* in RBCs were analyzed after ex vivo stimulation with dexamethasone or hydrocortisone, followed by 50 μg/mL poly (I:C), to assess whether prior exposure to glucocorticoids suppressed antiviral responses. Post-stress cortisol levels in fish can range from 20 to 500 ng/mL, depending on the species and stress intensity [32]. To determine the threshold at which immunosuppression occurs, three different concentrations of dexamethasone (1, 10, 100 μM) and four of hydrocortisone (20, 50, 100, 150 μM) within the designated physiological range were tested. Both dexamethasone and hydrocortisone similarly attenuated dsRNA-induced antiviral responses in RBCs, with dose-dependent immunosuppressive effects (Figure 4). Specifically, *mx1* expression was significantly suppressed following exposure to 100 μM dexamethasone (Figure 4A), while *isg15* expression was significantly suppressed by 20, 100 and 150 μM hydrocortisone (Figure 4B).

### Effects of dexamethasone on poly (I:C)-induced transcriptional responses of A. salmon red blood cells ex vivo analyzed by RNA-seq

A three-day exposure of RBCs to poly (I:C) induced 405 DEGs, while no genes were significantly downregulated compared to unstimulated controls (Figure 3A). Several upregulated genes were primarily associated with regulation of cellular homeostasis (“Response to stress”) and antiviral innate immune protection, including dsRNA-mediated signaling pathways, such as “RIG-I-like receptor” and “Herpes simplex virus 1 infection” (Figure 3B, left panel). Similar gene activation has previously been observed following a 24 h exposure of A. salmon RBCs to PRV-1 ex vivo [16], as well as during the early phase of PRV-1 infection (of A. salmon RBCs) in vivo [14].

When combined, dexamethasone and poly (I:C) resulted in 349 upregulated and 24 downregulated DEGs (Figure 3A). This is a decrease in the number of significantly upregulated DEGs compared to poly (I:C) alone (56 DEGs less).

**Figure 5:**
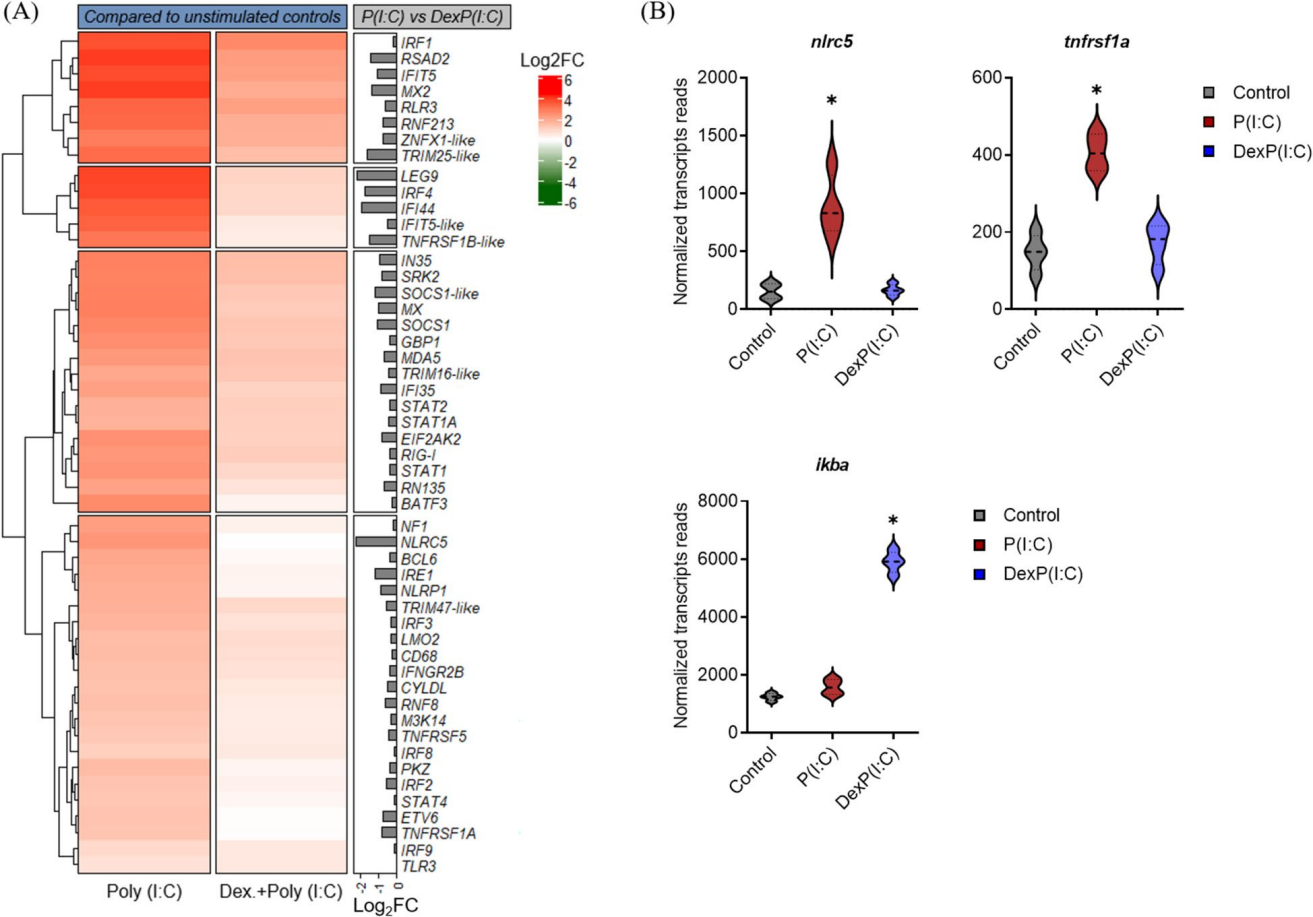
**Transcriptional analysis of A. salmon RBCs stimulated with poly (I:C) (50 μg/mL) or dexamethasone (100 μM) and poly (I:C), compared to unstimulated controls, based on RNA-seq data **(***n* = 4). A** **Gene expression profile in RBCs of A. salmon stimulated with either poly (I:C) alone or dexamethasone plus poly (I:C), compared to unstimulated controls. The heatmap includes Log**_**2**_**-fold change (Log**_**2**_**FC) of selected DEGs involved in “Immune system process” (GO:BP), as well as “RIG-I-like receptor”, “NOD-like receptor” and “Herpes simplex virus 1 infection” signaling pathways (KEGG), compared to unstimulated controls. Log**_**2**_**FC of selected DEGs is also provided from the comparison of poly (I:C) with dexamethasone plus poly (I:C)-stimulated RBCs (grey bar plot). Red: Higher expression level in stimulated RBCs compared to unstimulated controls; Green: Lower expression level in stimulated RBCs compared to unstimulated controls; White: No expression difference between stimulated and unstimulated RBCs. The darker the color, the stronger the regulation (higher or lower). B Violin plots showing selected genes involved in pathogen sensing by NOD-like receptors family (*nlrc5*** gene) and pro-inflammatory response (*tnfrsf1a* and *ikba* genes) with significantly different expression patterns between RBCs stimulated with poly (I:C) alone and those treated with dexamethasone and poly (I:C), compared to unstimulated controls. *p ≤ 0.01 in poly (I:C)-stimulated RBCs compared to unstimulated controls.

The expression patterns of selected poly (I:C)-induced DEGs involved in immune system processes (GO:BP, Figure 3B) and PRR-mediated antiviral pathways (KEGG pathways, Figure 3B), as well as how their responses to poly (I:C) were attenuated by dexamethasone, are shown in a heatmap (Figure 5A). Although the transcriptional levels of several antiviral genes, including *RIG-like receptor 3* (*rlr3*), *interferon (ifn)-induced protein with tetratricopeptide 5* (*ifit5-like*) and *viperin-like* (referred to as *rsad2*) genes, were reduced, their induction by poly (I:C) was not completely blocked. In addition, key mediators of antiviral response in A. salmon RBCs [14, 16], such as *tlr3* and *interferon regulatory factors (irfs) 1*, *3* and *9* genes, were significantly induced by poly (I:C), but unaffected by dexamethasone.

**Figure 6:**
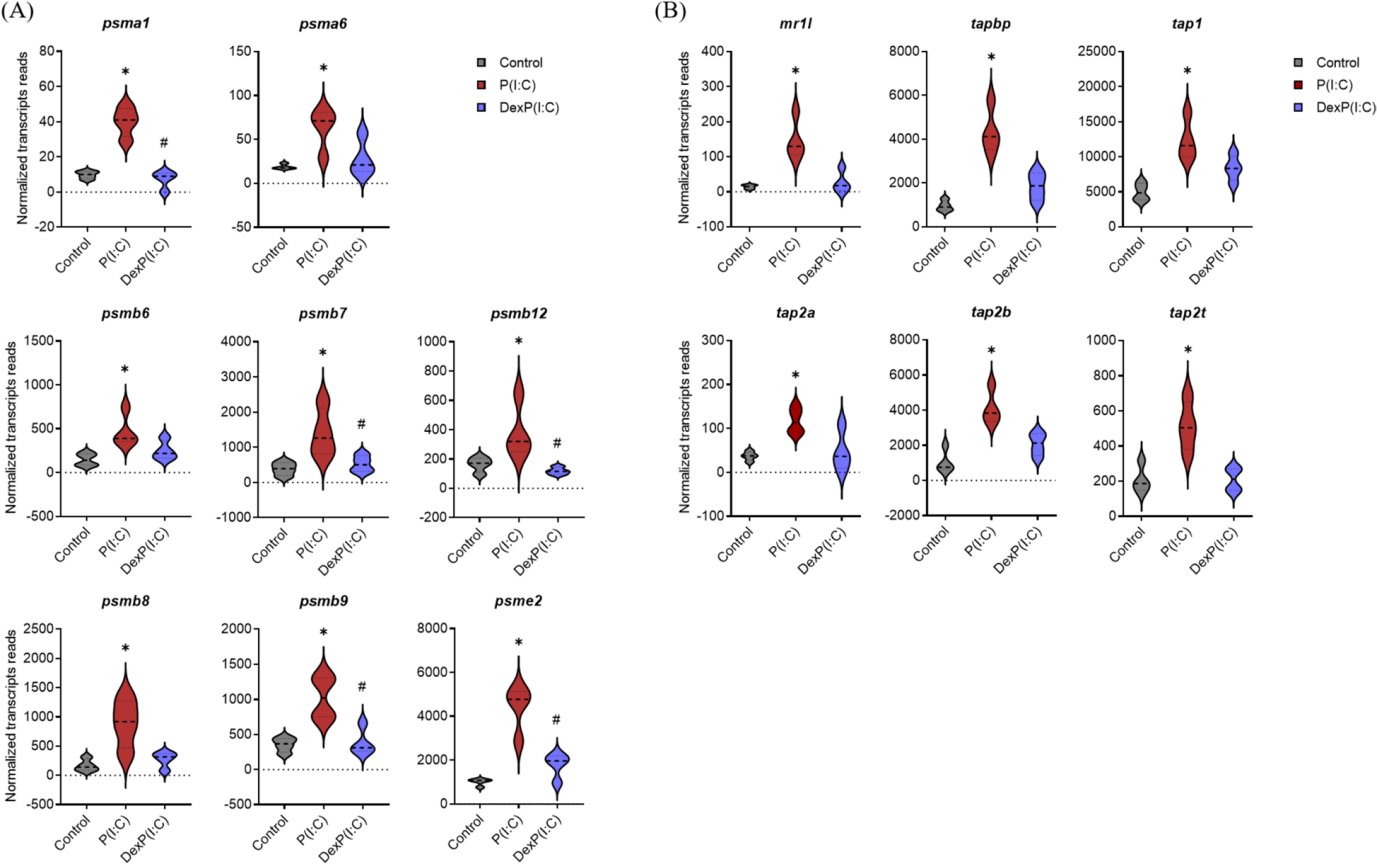
**Differential expression of selected genes associated with the formation of the proteasome apparatus, and antigen processing and presentation via MHC I in A. salmon RBCs, based on RNA-seq data **(***n* = 4).** **A** **Violin plots showing selected genes involved in proteasome formation and proteolytic degradation in RBCs stimulated with 50 μg/mL poly (I:C) alone or with 100 μM dexamethasone and 50 μg/mL poly (I:C), compared to unstimulated controls.** **B** Violin plots showing selected genes involved in MHC class I antigen processing and presentation in RBCs stimulated with poly (I:C) alone or with 100 μM dexamethasone and 50 μg/mL poly (I:C), compared to unstimulated controls. *p **≤ 0.01 in poly (I:C)-stimulated RBCs compared to unstimulated controls; # *p* ≤ 0.01 in dexamethasone and poly (I:C)-stimulated RBCs compared to poly(I:C) alone.**

Some genes implicated in the homeostatic control of innate immunity and antigen processing and presentation by major histocompatibility complex (MHC) class I, such as *NLR family CARD domain containing 5* (*nlrc5*), transporters *tap 1* and *2*, and *MHC class I-related gene protein* (*mr1*), were inhibited by dexamethasone (Figures 5B and 6B). Genes involved in regulation of cellular catabolic processes, proteolysis and protein modification were also significantly induced in response to poly (I:C) (Figure 3B, left panel). Among these DEGs were those encoding regulatory and core particles of 26S and 20S proteasomes, which mediate ubiquitin-dependent proteolysis and are involved in antigen processing and presentation, respectively (Figure 6). Dexamethasone significantly suppressed the expression of most of these genes, including *proteasome 20S (psm) subunit alpha 1* (*psma1*) and *psmb7*, *psmb9* and *psmb12* (Figure 6A). This suggests that hormonal stress responses may influence the proteolytic activity and MHC class I antigen processing and presentation following subsequent encounters with pathogens.

## Discussion

Environmental and physical stressors in aquaculture trigger cortisol release and subsequent GR signaling, facilitating physiological adaptations essential for maintaining internal homeostasis [33]. While cortisol measurement is an established method for assessing stress in fish [34], this approach may be limited in capturing the secondary effects of cumulative stressors, particularly due to impaired HPI sensitivity under allostatic overload [35, 36]. In this study, we demonstrated that A. salmon RBCs, which express GRs at high levels [14], responded to GCs both ex vivo and in vivo, and changes in the expression pattern of some genes, including *ddit4* and *fkbp5*, could serve as indicators of stress response in fish health assessment. However, it should be noted that the transcriptional responses of RBCs to dexamethasone ex vivo showed little resemblance to those induced in blood cells by cortisol injection in vivo. This discrepancy, which is discussed further below, may have arisen from the absence of complex systemic factors that regulate gene expression dynamics in a living organism, as opposed to the controlled conditions of cell studies [37].

Cortisol and dexamethasone are structurally similar compounds [38]. Dexamethasone is more stable than cortisol, with approximately twice the plasma half-life [39]. It also exhibits higher receptor binding affinity, and thereby higher potency than cortisol at equivalent doses [38]. In A. salmon RBCs, both dexamethasone and cortisol induced comparable transcriptional changes in genes involved in antiviral defense (e.g. *mx1* and *isg15*) and physiological stress responses (e.g. *fkbp5*) in a somewhat dose-dependent manner, as expected. Unlike mammals, most fish possess two GR genes (*gr1* and *gr2*), the activation of which may vary across tissues depending on circulating GC levels during a stress response [40–43]. Previous genetic analysis revealed four copies of GRs (two *gr1* located in chromosomes 4 and 13, and two *gr2* located in chromosomes 5 and 9) in the A. salmon genome [40]. This is consistent with our transcript findings, where all four *gr* variant genes were found to be highly expressed in RBCs. Structural and amino acid sequence differences among *gr* variants have previously been linked to divergences in protein–protein interactions and transcriptional regulation, leading to varying sensitivity to GC ligands and diverse functional roles across tissues [40]. For instance, *gr1* homologs exhibited delayed transcriptional activation in the head kidney compared to *gr2* genes, which were induced within 6 h following exposure to high stocking density stress. This delayed response of *gr1* was attributed to the lower cortisol levels during early stages of stress exposure [40]. While *gr* gene activation has been demonstrated following a 24 h glucocorticoid exposure in several tissues, little is known about their expression kinetics over time [40]. In this sense, the absence of significant differences in *gr* transcriptional profile between dexamethasone-stimulated RBCs and unstimulated controls may be attributed to the duration of glucocorticoid exposure and the timing of the analysis. Notably, *gr* expression levels in blood of A. salmon injected with cortisol in vivo remained at baseline at both time points, despite a pronounced cortisol peak observed at day two.

It has previously been reported that gilthead seabream (*Sparus aurata*) treated with cortisol showed higher accumulation of triglycerides in the liver, contrasting with increased glycogen storage in dexamethasone-treated fish [44]. Here, dexamethasone induced the overexpression of the *elovl4* gene, suggesting that GR-mediated signaling may influence lipid metabolism in RBCs. However, whether the activation of genes involved in LC-PUFA biosynthetic pathways [28] serves as a compensatory mechanism for the cells to meet the energy demands of stress responses, and/or whether it affects the regulation of other biological processes in RBCs, such as the innate immune response, remains to be explored.

Both dexamethasone and cortisol acted as potent modulators of the *fkbp5* gene in A. salmon RBCs, reinforcing its potential role in the stress response in fish [45]. Previous research on GR signaling in fish and mammalian models demonstrated that when cortisol levels are within the normal physiological range (resting state), the Fkbp5 protein interacts with the GR complex, hindering its translocation into the nucleus by reducing its affinity for GCs. Once the concentration of circulating GCs increases, Fkbp5 is degraded, allowing GR/GC complex to enter the nucleus and initiate the expression of target genes by binding to GR elements (GREs) [45, 46]. The *klf9* has been characterized as a key regulator of *fkbp5* activity and, consequently, GR-signaling during stress responses in zebrafish [45]. Here, the significantly high expression levels of *fkbp5* and *klf9* following dexamethasone stimulation, compared to their very low expression (close to zero transcripts detected) at resting state, suggest that RBCs may actively regulate their own stress responses.

The concentration of cortisol in the bloodstream of fish has been shown to increase within minutes to hours in response to stress [47], typically returning to baseline levels within 24 to 48 h post-exposure to the stressor [31, 48]. Interestingly, the relative expression of the *fkbp5* gene in dexamethasone-treated RBCs remained significantly high up to 14 days post-stimulation, compared to unstimulated controls. Thus, although plasma cortisol may be depleted within a few days after a stressful event, secondary stress responses may still be detectable. Notably, *fkbp5* induction was triggered even at very low GC doses, suggesting its high responsiveness to glucocorticoids, without a corresponding increase in gene mRNA levels at higher GC concentrations. Based on these findings, in scenarios where fish were exposed to consecutive stressors of varying intensities, *fkbp5* expression levels in blood would likely remain persistently high. Therefore, targeted transcriptional analysis of *fkbp5* alone may not be a sufficient indicator of the specific effects or intensity of the different stressors. This was evident when investigating transcriptional responses of *fkbp5* in whole blood of A. salmon two and four days post-cortisol injection, compared to untreated controls; despite variability in individual responses, mean *fkbp5* expression levels were not significantly different between control and cortisol-injected groups. Although differences in the experimental procedures (e.g. use of anesthesia) or the type of biological material used in each analysis (i.e. whole blood vs isolated RBCs) may contribute to transcriptional inconsistencies between the ex vivo and in vivo findings, the precise underlying factors are difficult to anticipate.

An intriguing observation was the upregulation of the *ddit4* gene in both the whole blood of cortisol-injected fish and ex vivo dexamethasone-stimulated RBCs. In mammals, *DDIT4* regulation, like that of *FKBP5* and *KLF9*, is directly modulated by GR-signaling [49]. Previous transcriptional analyses of mammalian skeletal muscle and blood cells have shown significant upregulation of *DDIT4* expression under hypoxic conditions and/or GC administration, supporting its characterization as a promising biomarker of the stress response [50, 51].

As reported by Thoen et al. [8] and Amundsen et al. [48] in the original trial, cortisol levels in injected fish were significantly higher on day two, but returned to baseline by day four. In A. salmon whole blood, *ddit4* overexpression was observed at two days post-cortisol injection, coinciding with peak cortisol levels. By day four, as cortisol levels declined, *ddit4* expression returned to basal levels. Similarly, *ddit4* upregulation was reported in RBCs four days post-stimulation with dexamethasone, compared to unstimulated controls. This dose–response relation between GC dynamics and *ddit4* regulation could serve as a reliable indicator for assessing both the onset and attenuation of stress response in fish.

Fish RBCs are immunologically active, engaging in diverse innate and adaptive immune processes in response to external stimuli [14, 52–55]. Their role as mediators of innate immunity was first demonstrated in rainbow trout RBCs following a 24 h exposure to poly (I:C), which triggered the expression of *tlr3*, *ifna* and *mx* immune genes [17]. Similar antiviral responses, indicative of dsRNA-mediated immunostimulation, were later described in A. salmon RBCs infected by the dsRNA virus PRV-1 in both ex vivo and in vivo studies [14, 16, 56]. Here, poly (I:C) served as a model to mimic the immune responses that typically occur during the acute phase of RBC infection by PRV-1 [14]. In particular, genes implicated in dsRNA recognition, such as *mda5* and *rlr-1/-3* [57], IFN-mediated transcriptional regulation (i.e. *irf* genes) and immune protection, were significantly induced following a three-day stimulation with poly (I:C). Genes involved in MHC class I antigen processing and presentation, like *psmb7* and *mr1*, were also upregulated, the expression of which has not previously been documented in A. salmon RBCs. Despite the consistent effects of poly (I:C) on the gene expression profile of treated cells, the responses among individuals varied substantially. This may be due to differences in RBC maturation stage, with senescent cells displaying reduced reactivity compared to “younger” cells [14, 58].

Chronic stress exerts negative effects on innate immune function by promoting prolonged cortisol release through the HPI-axis and stimulating GR/GC-signaling pathways [7, 59]. In fish, stress-induced immunosuppression has been linked to the overexpression of anti-inflammatory mediators, such as *NF-kB inhibitor alpha protein* (*nfkbiaa* or *ikba*), which inhibits IFN production by disrupting NF-kB transcriptional regulator activity [7, 60]. Activation of GR/GC-signaling pathway in A. salmon RBCs by dexamethasone significantly upregulated *ikba*, which may underlie the attenuated responsiveness of some IFN-stimulated antiviral effectors (e.g. *rsad2* and *mx2*) to poly (I:C) through repression of NF-kB and IFN signaling. The decreased expression levels of some cytokine receptor genes, such as *tumor necrosis factor receptor superfamily member 1A* (*tnfrsf1a*), which interacts with secreted Tnf-a, to induce innate immune response [61], may further support this hypothesis. In mammals, *NF-kB* has also been shown to interact with *FKBP5*, modulating the antiviral immune response [46, 62, 63]. Specifically, *FKBP5* was found to promote *IKBA* degradation, thereby enhancing downstream cytokine production through NF-kB signaling [63]. However, in the present study, the role of *fkbp5* in the regulation of innate immunity in fish RBCs was not established.

Among the PRRs responding to poly (I:C), only the expression of the *nlrc5* gene was entirely blocked by GCs, returning to basal levels. A previous study investigated the *nlrc5* transcriptional profile in several tissues of A. salmon at parr and smolt stages, and linked its downregulation to cortisol elevation during smoltification [64]. While GCs clearly affected the transcriptional regulation of innate immune genes in RBCs, specific molecular interactions between the endocrine and immune systems in this context remain poorly understood and warrant further investigation.

Poly (I:C) induced the expression of several MHC class I antigen and proteasome subunit genes, an effect that was entirely blocked (down to baseline levels) by dexamethasone. The impairment of antigen processing and presentation by glucocorticoids has previously been demonstrated in mammalian dendritic cells through transcriptional and functional analyses [65]. In particular, GR pathway activation prior to infection has been linked to reduced generation of antigenic peptides by disrupting the activity of proteolytic molecules essential for degradation of viral proteins [65]. In salmonids, cortisol injection prior to IPNV infection resulted in significant downregulation of MHC class I gene in mucosal and lymphoid tissues [66]. While the exact mechanism in fish is not fully elucidated, suppression of genes encoding proteasome subunits, such as *psmb-6–9*, and MHC-I regulatory proteins, such as *TAP-binding protein* (*tapbp*), which mediates antigen peptide transport across the endoplasmic reticulum (ER) [67], may reduce the capacity of RBCs to present viral peptides to the immune system. This complex interplay between hormonal stress responses, compromised antiviral immunity, and reduced antigen presentation may create conditions that favor viral replication and dissemination across tissues. This is in line with observations in trials where fish infected by PRV-1 and SGPV exhibited reduced robustness and increased mortality when exposed to stress [8, 11].

In conclusion, A. salmon RBCs may play a critical role in physiological adaptations to stress, as they respond to cortisol and dexamethasone, modulating their immune and metabolic responses. The *fkbp5* and *klf9* genes may play a role in the secondary effects of stress in RBCs, based on their strong transcriptional upregulation by GR agonists ex vivo. In cortisol-injected fish, the *ddit4* gene, regulation of which appeared to be GC-dependent both ex vivo and in vivo, may emerge as a promising biomarker candidate. Exposure of RBCs to GCs prior to stimulation with poly (I:C) resulted in the attenuation of several genes involved in IFN-mediated immunity and significantly suppressed genes involved in proteolytic degradation and MHC class I antigen processing and presentation. Stress-mediated dysregulation of antiviral immune function and immunological memory may reduce both short- and long-term immune protection against viruses, and increase the susceptibility of previously stressed fish to subsequent infections.

## Supplementary Information

**Additional file 1 Principal Component Analysis – RBCs (***n*** = 5) ex vivo stimulation.** Original principal component analysis for Atlantic salmon red blood cells (RBCs) (n = 5) treated with 100 μM dexamethasone (four days), 50 μg/mL poly (I:C) (three days), dexamethasone and poly (I:C) together, and untreated controls. Due to strong deviation of one control sample, this entire experiment was omitted from further analysis.

**Additional file 2 Principal Component Analysis – RBCs (***n*** = 4) ex vivo stimulation.** Principal component analysis for Atlantic salmon red blood cells (RBCs) (n = 4) treated with 100 μM dexamethasone (four days), 50 μg/mL poly (I:C) (three days), dexamethasone and poly (I:C) together, and untreated controls. RNA-seq data from these experiments are further analyzed in the present manuscript.

**Additional file 3 Principal Component Analysis – Whole blood in vivo**. Principal component analysis for whole blood of Atlantic salmon two and four days post-injection (2d and 4d, respectively) with cortisol, and non-cortisol injected controls.

**Additional file 4 Transcriptional responses of RBCs to 100 μM dexamethasone**. The lists of all upregulated DEGs in response to dexamethasone.

**Additional file 5 Transcriptional responses of RBCs to 100 μM dexamethasone.** The lists of all downregulated DEGs in response to dexamethasone.

**Additional file 6 Transcriptional responses of whole blood cells to cortisol.** The lists of all upregulated DEGs in response to cortisol, two days post-injection.

**Additional file 7 Transcriptional responses of whole blood cells to cortisol.** The lists of all downregulated DEGs in response to cortisol, two days post-injection.

**Additional file 8 Transcriptional responses of whole blood cells to cortisol.** The lists of all upregulated DEGs in response to cortisol, four days post-injection.

**Additional file 9 Transcriptional responses of whole blood cells to cortisol.** The lists of all downregulated DEGs in response to cortisol, four days post-injection.

**Additional file 10 Antiviral responses in Atlantis salmon red blood cells (RBCs) treated with 50 μg/mL poly (I:C). **The expression levels of mx1 and isg15 were measured by RT-qPCR at three samplings points, one-, three- and seven- days post exposure to poly(I:C). The expression levels in stimulated RBCs relative to the unstimulated controls were calculated for each sample (*n* = 3). Error bars represent standard deviation in each plot. *: *p* < 0.05; **: *p* < 0.01.

## Data Availability

The data generated and analyzed in the current study are available in NCBI SRA BioProjects PRJNA1042786 and PRJNA1042788.
