# Supplementary figures and images for: Effects of glucocorticoid receptor activation on gene expression and antiviral responses in Atlantic salmon (Salmo salar L.) red blood cells
Source: Vet Res. 2025 Oct 7;56:188. doi: 10.1186/s13567-025-01605-w (PMC12505585; doi:10.1186/s13567-025-01605-w)

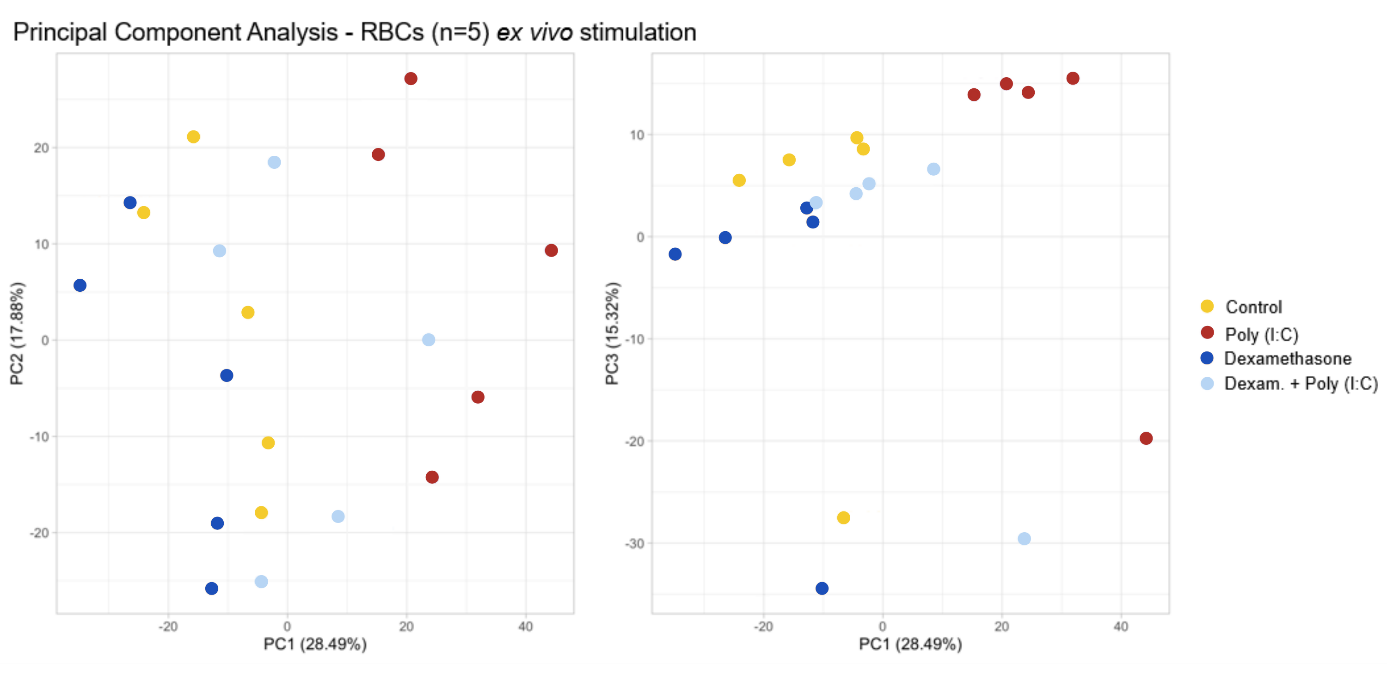

Supplement: Supplementary file 1 — Additional file 1 Principal Component Analysis – RBCs (n = 5) ex vivo stimulation. Original principal component analysis for Atlantic salmon red blood cells (RBCs) (n = 5) treated with 100 μM dexamethasone (four days), 50 μg/mL poly (I:C) (three days), dexamethasone and poly (I:C) together, and untreated controls. Due to strong deviation of one control sample, this entire experiment was omitted from further analysis. [file 13567_2025_1605_MOESM1_ESM.docx]

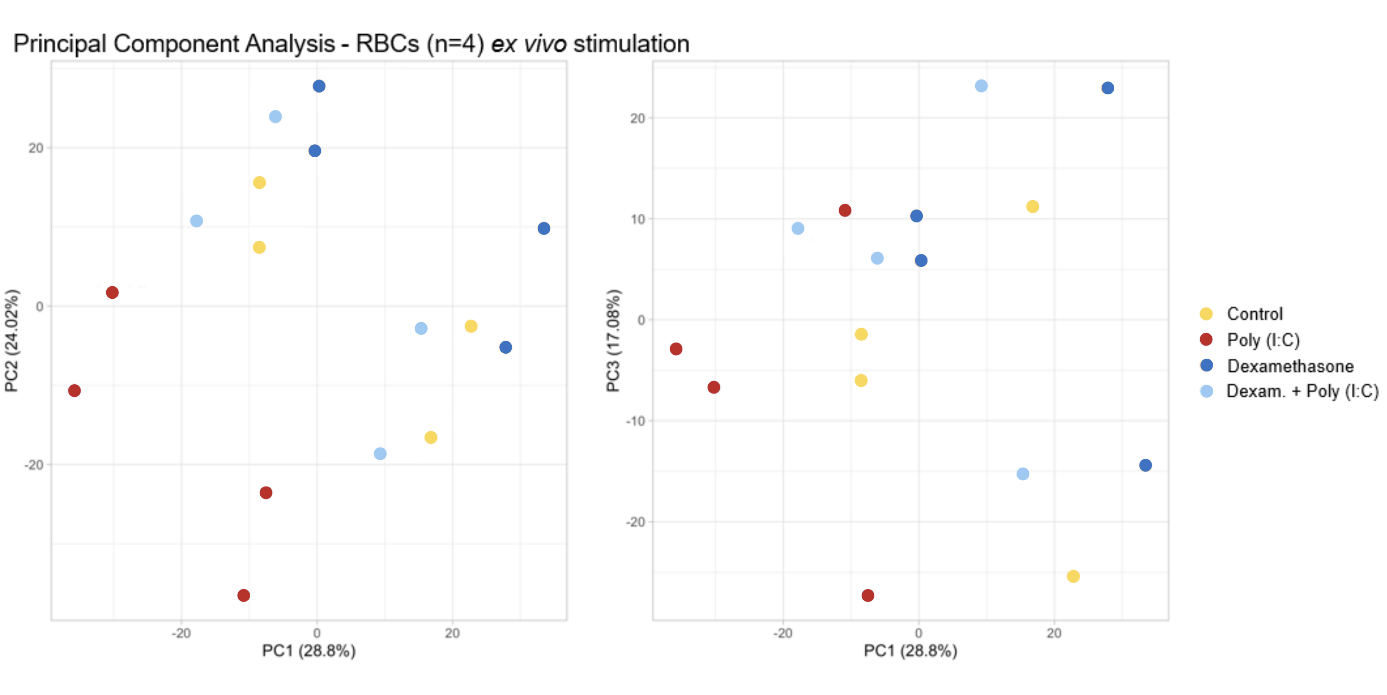

Supplement: Supplementary file 2 — Additional file 2 Principal Component Analysis – RBCs (n = 4) ex vivo stimulation. Principal component analysis for Atlantic salmon red blood cells (RBCs) (n = 4) treated with 100 μM dexamethasone (four days), 50 μg/mL poly (I:C) (three days), dexamethasone and poly (I:C) together, and untreated controls. RNA-seq data from these experiments are further analyzed in the present manuscript. [file 13567_2025_1605_MOESM2_ESM.docx]

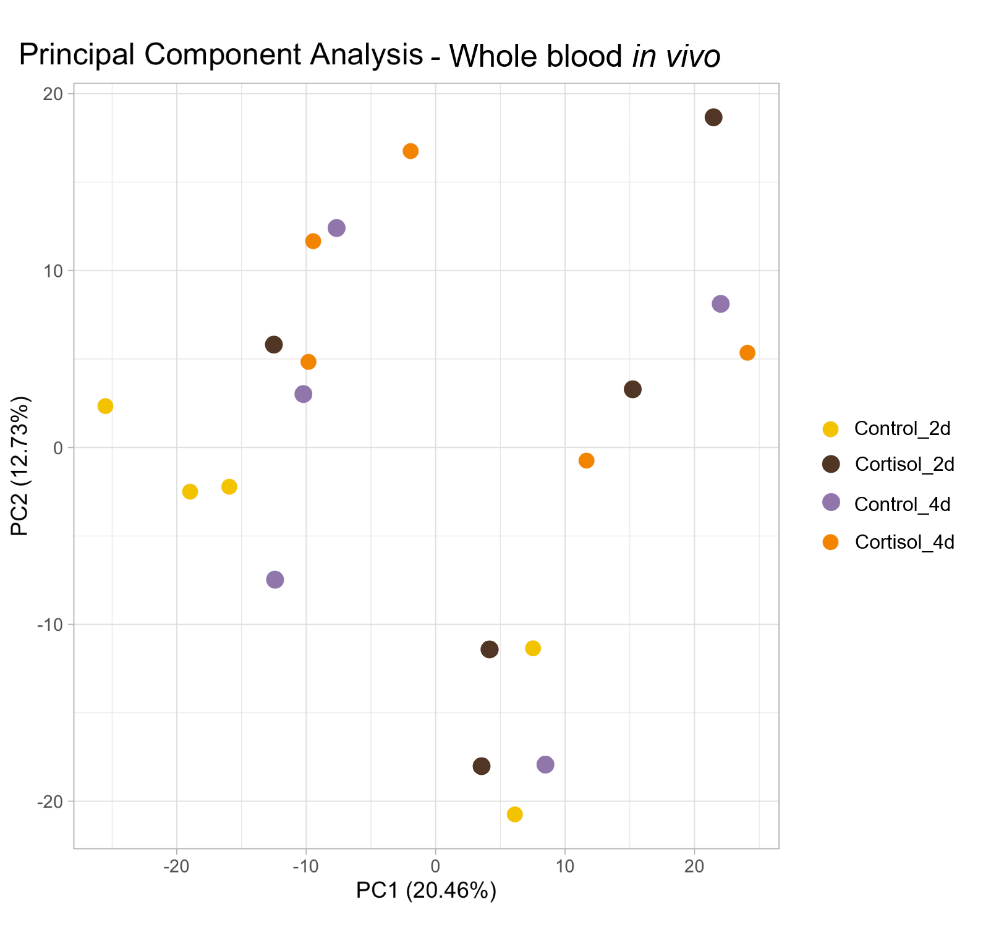

Supplement: Supplementary file 3 — Additional file 3 Principal Component Analysis – Whole blood in vivo. Principal component analysis for whole blood of Atlantic salmon two and four days post-injection (2d and 4d, respectively) with cortisol, and non-cortisol injected controls. [file 13567_2025_1605_MOESM3_ESM.docx]

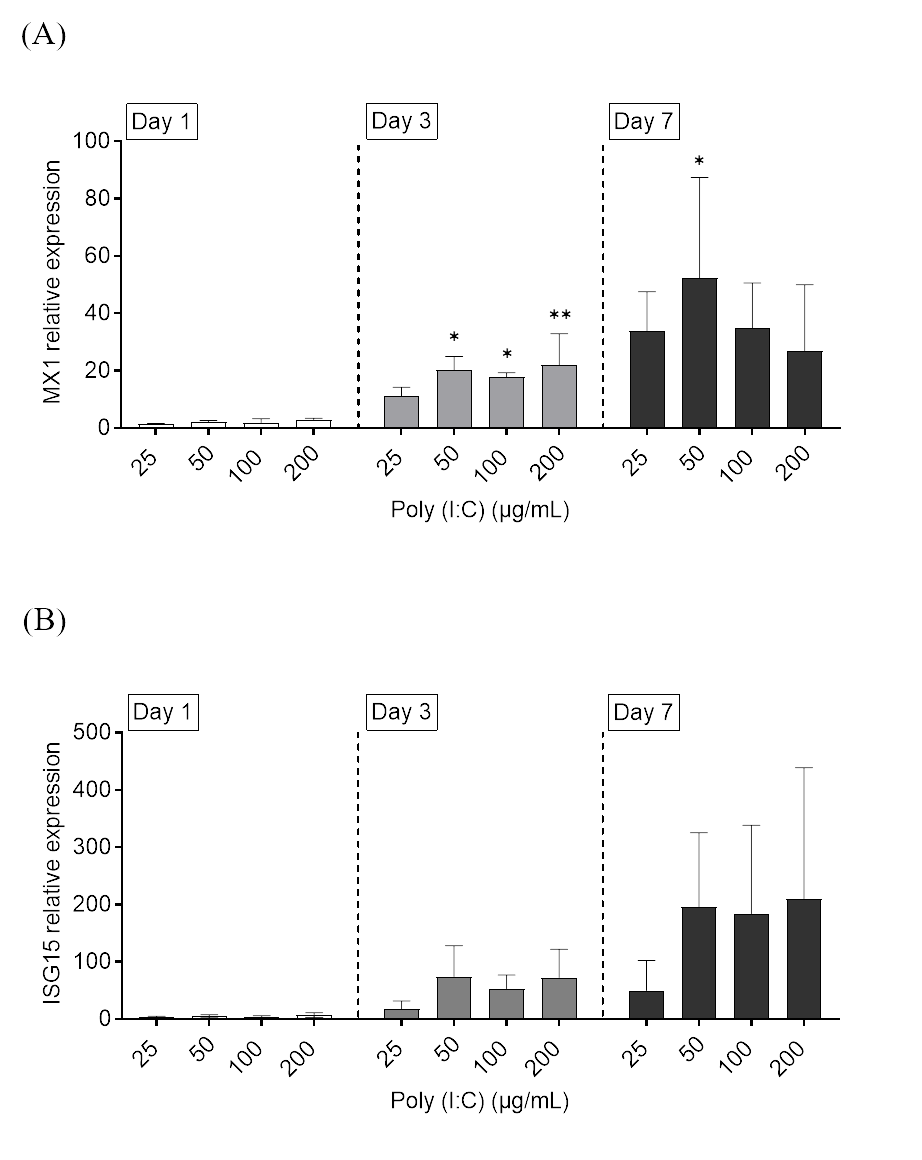

Supplement: Supplementary file 10 — Additional file 10 Antiviral responses in Atlantis salmon red blood cells (RBCs) treated with 50 μg/mL poly (I:C). The expression levels of mx1 and isg15 were measured by RT-qPCR at three samplings points, one-, three- and seven- days post exposure to poly(I:C). The expression levels in stimulated RBCs relative to the unstimulated controls were calculated for each sample (n = 3). Error bars represent standard deviation in each plot. *: p < 0.05; **: p < 0.01. [file 13567_2025_1605_MOESM10_ESM.docx]
